# Measuring the cost-effectiveness of treatments for people with multiple sclerosis: Beyond quality-adjusted life-years

**DOI:** 10.1177/1352458520954172

**Published:** 2020-09-03

**Authors:** Annie Hawton, Elizabeth Goodwin, Kate Boddy, Jennifer Freeman, Sarah Thomas, Jeremy Chataway, Colin Green

**Affiliations:** Health Economics Group, University of Exeter Medical School, University of Exeter, Exeter, UK/NIHR Applied Research Collaboration (ARC) South West Peninsula, University of Exeter Medical School, University of Exeter, Exeter, UK; Health Economics Group, University of Exeter Medical School, University of Exeter, Exeter, UK; NIHR Applied Research Collaboration (ARC) South West Peninsula, University of Exeter Medical School, University of Exeter, Exeter, UK; School of Health Professions, Faculty of Health, University of Plymouth, Plymouth, UK; Faculty of Health and Social Sciences, Bournemouth University, Bournemouth, UK; Queen Square Multiple Sclerosis Centre, Department of Neuroinflammation, UCL Queen Square Institute of Neurology, Faculty of Brain Sciences, University College London, London, UK/National Institute for Health Research, University College London Hospitals, Biomedical Research Centre, London, UK; Health Economics Group, University of Exeter Medical School, University of Exeter, Exeter, UK/Collaboration (ARC) South West Peninsula, University of Exeter Medical School, University of Exeter, Exeter, UK

**Keywords:** Multiple sclerosis, cost-effectiveness, quality-adjusted life-years, cost-effectiveness analysis, health-related quality of life, quality of life

## Abstract

**Background:**

It is a familiar story. A promising multiple sclerosis (MS) treatment clears the three regulatory hurdles of safety, quality and efficacy, only to fall at the fourth: cost-effectiveness. This has led to concerns about the validity of the measures typically used to quantify treatment effects in cost-effectiveness analyses and in 2012, in the United Kingdom, the National Institute for Health and Care Excellence called for an improvement in the cost-effectiveness framework for assessing MS treatments.

**Objective and Methods:**

This review describes what is meant by cost-effectiveness in health/social care funding decision-making, and usual practice for assessing treatment benefits.

**Results:**

We detail the use of the quality-adjusted life-year (QALY) in resource allocation decisions, and set out limitations of this approach in the context of MS.

**Conclusion:**

We conclude by highlighting methodological and policy developments which should aid addressing these limitations.

## Introduction

It is a familiar story. A promising treatment for people with multiple sclerosis (MS) clears the three regulatory hurdles of safety, quality and efficacy, only to fall at the fourth: cost-effectiveness. For the past two decades, from disease-modifying drugs, such as the interferons, to symptomatic treatments, like fampridine and sativex, the pattern has been the same. Clinicians and patients feel the treatment provides benefits, but the National Institute for Health and Care Excellence (NICE) states the benefits are not worth the costs, and it will not be funded by the National Health Service (NHS). This has led to concerns about the relevance and validity of the measures typically used to quantify treatment effects in cost-effectiveness analyses^1^ and, in 2012, NICE called for an improvement in the cost-effectiveness framework for assessing treatments for people with MS.^2^

This review describes what is meant by cost-effectiveness in health/social care funding decision-making, and usual practice for assessing treatment benefits. We set out limitations of this approach, with illustrations in the MS context, and conclude by highlighting methodological and policy developments to address such limitations.

## Cost-effectiveness

The public funding of health/social care interventions largely depends on demonstrating cost-effectiveness. This is assessed by weighing the additional costs of treatments against the additional benefits they provide.^3^ In the United Kingdom^4^ and other national health policy contexts, for example, Germany, the Netherlands, Australia and Canada, cost-effectiveness analyses include estimating the cost-per-QALY (quality-adjusted life-year) of interventions.

## QALYs

The QALY combines length and quality of life in a single outcome measure. Each year of life is weighted by quality of life during that time. Quality of life is represented by QALY weights on a scale from zero (equivalent to being dead) to one (perfect health). QALY weights can also be negative, representing quality of life thought worse than being dead. A higher number of QALYs indicates a better health outcome.

An advantage of the QALY is its applicability to a variety of conditions and interventions, providing a common metric to compare cost-effectiveness. This largely explains its appeal for informing system-wide funding decisions.^4–7^

## QALY weights and preference-based measures

The quality of life ratings used as QALY weights are most commonly obtained from an existing preference-based measure (PBM) of health-related quality of life (HRQoL), such as the EQ-5D^8^ or the SF-6D.^9^ PBMs have two components:

1. A descriptive system – This describes an individual’s health on a number of dimensions (e.g. mobility, pain), each with a number of severity levels. Each combination of levels/dimensions constitutes a ‘health state’. These descriptive systems (e.g. the EQ-5D) are often completed by individuals in clinical trials.
2. QALY weights for health states – Tariffs of QALY weights pre-exist for commonly used PBMs, for example, a tariff of QALY weights was obtained for the health states that the EQ-5D describes via a representative sample of the UK general population. The tariff is used to apply QALY weights to EQ-5D health states reported by individuals in clinical trials and defined in cost-effectiveness models.^8^

QALY weights are usually derived by eliciting people’s preferences for a sub-group of the descriptive system’s health states (and statistically modelling preferences for the remaining states). Preferences are obtained using a variety of methods which provide weights on the zero to one scale.

## QALY weights and MS

QALY weights can have a major impact on cost-effectiveness results,^10^ but there are inconsistencies and marked variability in reported weights.^11^ For example, the UK MS Survey^12^ reported higher (i.e. better) EQ-5D scores at Expanded Disability Status Scale (EDSS) score 4 (‘Relatively severe disability’) compared to EDSS 3 (‘Moderate disability’). These weights have been used in NICE appraisals of ocrelizumab, dimethyl fumarate, fingolimod and natalizumab. In addition, the QALY weight decrement of 0.071 associated with a relapse^12^ has been widely used, but decrements of between 0.029^13^ and 0.8^14^ have also been cited.

## Characteristics of QALY weights

Three key areas of debate surround the current QALY approach: how to describe health states, whose preferences to use, and the scope of the health state description. These concerns are relevant to the validity of the QALY approach to MS.

### Generic PBMs

The QALY weights most frequently used are from *generic* PBMs, designed to be maximally suitable for various conditions/interventions. Internationally, the EQ-5D is the most frequently used^8^ and is specified for use in NICE’s ‘reference case’.^4^

### General population preferences

Typically, QALY weights are elicited from members of the *general population*, the approach recommended in many policy settings, for example, NICE^4^ and the Panel on Cost-Effectiveness in Health and Medicine.^3^ This is based on the argument that in publicly-funded healthcare systems societal preferences should guide resource allocation to reflect the views of those funding the service.

### HRQoL

QALY weights are based on people’s *health state preferences*, for example, the EQ-5D includes the dimensions mobility, usual activities, pain/discomfort, self-care and anxiety/depression. When QALY weights are applied to these health states, the instruments are described as measuring *health-related quality of life*.

## Potential limitations of the QALY for MS

### Generic PBMs

Relevance and responsiveness are key properties of any measure. Relevance concerns an instrument’s coverage of domains central to the measurement construct and important to the population, and is typically assessed by exploring content, convergent and discriminative validity. Convergent validity is the relationship with other measures that assess the same construct. Discriminative validity is the ability to distinguish between groups known to differ in the construct of interest.^15^ Responsiveness is an instrument’s ability to detect changes in the construct it measures. A measure with poor responsiveness may fail to capture benefits (or harms) of a treatment.

The relevance of the content of generic PBMs to MS has been questioned.^12,16^ A recent systematic review^16^ concluded that the content validity of the EQ-5D and the SF-6D is poor, largely due to omission of domains relating to fatigue (EQ-5D), mobility (SF-6D) and cognition (both measures). A lack of convergent validity of generic PBMs with other HRQoL measures in relation to MS has also been described.^16^ Some research suggests that the EQ-5D and SF-6D distinguish between degrees of MS disability, while other work indicates a limited ability to capture changes in HRQoL across disease severity. Concerns have also been raised regarding the responsiveness of these generic PBMs to illness-related events and treatment effects. To date, no MS papers have been identified which explore the responsiveness of PBMs.^16^

### General population preferences

Basing QALY weights on general population preferences does not take specific account of the preferences of people with MS. Two main arguments support the use of *patient* preferences in cost-effectiveness analyses.^17^ The first relates to welfare economics, which posits that the well-being of society equals the sum of the well-being of its individual members. This implies that decisions regarding public funding should be based on the preferences of those set to gain/lose directly from decisions made, rather than the wider population who may be unaffected. The second is that people with MS live with the condition and are better placed to assess how it affects quality of life.

Qualitative research suggests differences in the rationales of people with MS and the general public for their health state preferences,^18^ and significant differences have been identified between QALY weights from people with MS and the general population.^19^

### HRQoL

The particular conceptualisation of HRQoL used in relation to PBMs differs from its broader conceptualisation. HRQoL has been defined as, ‘the patient’s subjective perception of the impact of his [*sic*] disease and its treatment(s) on his [*sic*] daily life, physical, psychological and social functioning and well-being’.^20^ In the framework of PBMs, HRQoL is operationalised as the preference weight assigned to an individual’s health state based (usually) on general population preferences for that health state. A change in a PBM score indicates a change in health status, represented by the associated change in preference weight.

Treatments for people with MS can have effects beyond health and HRQoL as captured by PBMs. Interventions may have broader individual and/or societal impacts, for example, engagement with community activities, employment. Despite the significance of these wider impacts, they have been under-represented in cost-effectiveness analyses of MS treatments.^11^ The discipline of health economics is founded on a societal perspective,^3^ but the policy focus on the QALY has steered emphasis away from the effects of interventions on broader aspects of quality of life and well-being. As such, treatment effects may be missed and cost-effectiveness analyses may not reflect the true impact of MS treatments on people’s lives.^1,11^

## Beyond the QALY and cost-effectiveness analyses of treatments for MS

Here we describe how each of these aforementioned QALY issues may be addressed in the context of MS.

### Development of MS-specific PBMs and QALY weights

There is a tension in the use of generic PBMs. They must be sufficiently generic to apply to multiple conditions and interventions, and relevant to particular illnesses. The challenge of finding this balance has led to the development of condition-specific PBMs.

Condition-specific PBMs comprise a descriptive system tailored to a particular illness, thus offering greater potential responsiveness. Three MS-specific PBMs have been developed.^21–23^ The Preference-Based Multiple Sclerosis Index (PBMSI)^21^ is based on preferences elicited using a rating scale to produce a scoring algorithm. The Multiple Sclerosis Impact Scale-Preference Based Measure (MSIS-PBM)^22^ and the Multiple Sclerosis Impact Scale-Eight Dimensions (MSIS-8D)^23^ are based on responses to the Multiple Sclerosis Impact Scale-29 (MSIS-29). This measures the effect of MS on HRQoL and is commonly used in clinical trials. These latter measures enable QALY weights to be estimated from patient-level MSIS-29 data and facilitate retrospective analyses using existing data.^24^

The use of condition-specific PBMs to provide QALY weights has generated considerable debate.^25^ Some argue that to compare the results of cost-effectiveness analyses, the same descriptive system must be used to assess outcomes; others^26^ suggest results are comparable providing the same preference elicitation methods are used. As such, health state preferences for the MSIS-PBM^22^ and the MSIS-8D^23^ were elicited using NICE recommended methods.^4^

Using a condition-specific rather than a generic PBM involves a trade-off between the advantages/disadvantages of the measures in relation to the condition of interest.^27^ For MS, the potential limitations of generic PBMs support the use of a condition-specific PBM, and there is some evidence that these are more sensitive to differences across the range of HRQoL than generic PBMs.^23^

### QALY weights from people with MS

Neither the use of ‘patient’ or ‘public’ preferences to inform resource allocation decisions can claim superior theoretical or empirical validity. However, within health policy contexts that are increasingly patient-centred,^28^ it seems pertinent to consider the role of patient preferences in cost-effectiveness analyses.

The PBMSI^21^ is based on preferences of people with MS, and the MSIS-8D has tariffs of public^29^ and patient^30^ QALY weights. Comparison of the MSIS-8D public and patient tariffs has highlighted differences. People with MS placed greater value on the health states than did the general population, a difference which was significant regardless of the severity of the health states. The general population placed greater importance on depression, fatigue and daily activities, and people with MS placed greater importance on cognition. These findings suggest the impact of using patient rather than public QALY weights on the results of cost-effectiveness analyses will vary, depending on the specific dimensions of HRQoL affected by the intervention assessed, for example, interventions targeting cognition may appear more cost-effective if assessed using QALY weights from people with MS.^19^ The choice of ‘whose preferences’ to use when estimating QALYs could have important consequences for reimbursement decisions.^19^

Cost-effectiveness based on patient preferences could be considered in conjunction with results based on population preferences, yet NICE currently stipulates that QALY weights should be based on general population preferences.^4^ Given the central role of cost-effectiveness analyses in resource allocation decisions, it seems judicious to find additional ways of building what is relevant and important to people with MS into such research. The need for meaningful public and patient involvement (PPI) is indicated. Patients are experts at providing insight into the lived experience of a condition, as clinicians are experts at providing an overview of a disease, and health economists are experts in the methodology of cost-effectiveness analyses.

Input from people with MS when developing the MSIS-8D^31^ resulted in the removal of several implausible health states from the preference elicitation survey. This is likely to have improved the validity of the QALY weights and enabled the MSIS-8D to provide a better indication of treatment impacts. Discussion of PPI in health economics research is growing, and our experiences of working with people with MS suggest involvement can be meaningful and productive in informing QALY developments.

### Beyond HRQoL

In 2013, NICE’s remit was extended from making funding recommendations about healthcare interventions to producing social care guidance.^26^ This has led to increased scrutiny of the dominance of the health-related QALY,^32^ and a greater focus in health/social care policy on measuring broader benefits. NICE^26^ and the Social Care Institute for Excellence (SCIE)^33^ have recommended that evaluations of interventions with a social care element should include their impact on ‘well-being’.

Well-being refers to being able to do and be the things in life that matter to individuals.^34^ This seems particularly relevant to MS, given its wide-ranging impact and the breadth of interventions which may help, for example, rehabilitation programmes to support return to work, home adaptations and personal care. NICE now recommends the ICEpop CAPability measure for Adults (ICECAP-A)^31^ and the Adult Social Care Outcomes Toolkit (ASCOT);^35^ measures that extend beyond health to capture this wider construct. The ICECAP-A focuses on attachment, security, role, enjoyment and control. The ASCOT measures social care-related quality of life and includes: control over daily life; personal cleanliness and comfort; food and drink; personal safety; social participation and involvement; occupation; accommodation and dignity. Both measures have a tariff of ‘well-being weights’ (akin to QALY weights), which indicate people’s preferences for the well-being states they describe. They are yet to be used in cost-effectiveness analyses of MS treatments, and their relevance and responsiveness need to be assessed in relation to MS.

## Conclusion

The way that treatment benefits are currently captured for health policy decision-making has limitations in relation to MS. Generic PBMs may be limited in their relevance to MS and to fluctuations in the illness; the use of general public health state preferences may miss the lived experience of MS; and the operationalisation of HRQoL based on PBMs may not detect broader treatment impacts.

Going beyond the established QALY approach is timely, given recent and unfolding methodological and policy developments. MS-specific QALY measures are available for use alongside generic PBMs, with patient, as well as general public, tariffs. PPI in MS cost-effectiveness outcome assessment is developing, and NICE’s acceptance of well-being measures provides an opportunity to capture wider treatment impacts.

The collaboration of health economists, clinicians, people with MS, and those supporting people with MS, to develop the methods used for measuring the benefits of treatments in cost-effectiveness analyses, and to influence the resource allocation policy framework seems key. Since the 1990s, huge progress has been made in relation to the third regulatory hurdle of efficacy, with the advent of, and engagement with, evidence-based medicine. A joint enterprise addressing the fourth hurdle of cost-effectiveness should help facilitate its successful negotiation.
